# Integrating restriction site-associated DNA sequencing (RAD-seq) with morphological cladistic analysis clarifies evolutionary relationships among major species groups of bee orchids

**DOI:** 10.1093/aob/mcx129

**Published:** 2018-01-09

**Authors:** Richard M Bateman, Gábor Sramkó, Ovidiu Paun

**Affiliations:** 1 Jodrell Laboratory, Royal Botanic Gardens Kew, Richmond, Surrey, UK; 2 Department of Botany, University of Debrecen, Egyetem, Debrecen, Hungary; 3 MTA-DE ‘Lendület’ Evolutionary Phylogenomics Research Group, Egyetem, Debrecen, Hungary; 4 Department of Botany and Biodiversity Research, University of Vienna, Rennweg, Vienna, Austria

**Keywords:** Biogeography, character mapping, coalescence, convergence, evolution, internal transcribed spacer, macrospecies, Mediterranean, microspecies, morphology, *Ophrys*, paedomorphosis, phylogenetics, plastid, pseudo-copulation, RAD-seq, systematics

## Abstract

**Background and Aims:**

Bee orchids (*Ophrys*) have become the most popular model system for studying reproduction via insect-mediated pseudo-copulation and for exploring the consequent, putatively adaptive, evolutionary radiations. However, despite intensive past research, both the phylogenetic structure and species diversity within the genus remain highly contentious. Here, we integrate next-generation sequencing and morphological cladistic techniques to clarify the phylogeny of the genus.

**Methods:**

At least two accessions of each of the ten species groups previously circumscribed from large-scale cloned nuclear ribosomal internal transcibed spacer (nrITS) sequencing were subjected to restriction site-associated sequencing (RAD-seq). The resulting matrix of 4159 single nucleotide polymorphisms (SNPs) for 34 accessions was used to construct an unrooted network and a rooted maximum likelihood phylogeny. A parallel morphological cladistic matrix of 43 characters generated both polymorphic and non-polymorphic sets of parsimony trees before being mapped across the RAD-seq topology.

**Key Results:**

RAD-seq data strongly support the monophyly of nine out of ten groups previously circumscribed using nrITS and resolve three major clades; in contrast, supposed microspecies are barely distinguishable. Strong incongruence separated the RAD-seq trees from both the morphological trees and traditional classifications; mapping of the morphological characters across the RAD-seq topology rendered them far more homoplastic.

**Conclusions:**

The comparatively high level of morphological homoplasy reflects extensive convergence, whereas the derived placement of the *fusca* group is attributed to paedomorphic simplification. The phenotype of the most recent common ancestor of the extant lineages is inferred, but it post-dates the majority of the character-state changes that typify the genus. RAD-seq may represent the high-water mark of the contribution of molecular phylogenetics to understanding evolution within *Ophrys*; further progress will require large-scale population-level studies that integrate phenotypic and genotypic data in a cogent conceptual framework.

## INTRODUCTION

Orchidaceae is arguably Europe’s most charismatic plant family and *Ophrys* (bee orchids) is unquestionably Europe’s most charismatic orchid genus. Its fascination for professional botanists and amateur natural historians alike derives primarily from its remarkable pseudo-copulatory reproductive syndrome, which is best viewed as a form of parasitism (e.g. Vereecken, 2009); the orchid deceives naïve male insects into attempting to mate with its flowers, occasionally transferring pollinaria to the male insect which are then (very occasionally) deposited on the stigma of another conspecific flower when the insect repeats its misconceived sexual advances (a system reputedly first comprehended by Pouyanne, 1917). Although this syndrome has since been identified in a few other orchid lineages (e.g. Blanco and Barboza, 2005; Swarts *et al.*, 2014), the spectrum of accumulated presumed adaptations is both especially impressive and especially well understood in *Ophrys*.

Responsibility for attracting the male insects to bee orchid flowers falls almost entirely on a single phenotypically complex petal, the labellum, which brings three categories of weaponry to bear on the hapless insect: in chronological order they are scent, sight and touch. First the potential pollinator detects a remarkable cocktail of pseudo-pheromones (e.g. Ayasse *et al.*, 2000; Schiestl and Cozzolino, 2008; Stökl *et al.*, 2008; Gögler *et al.*, 2009; Breitkopf *et al.*, 2013; Sedeek et al., 2013, 2014, 2016; Vereecken and Francisco, 2014), then it observes the visual cues provided by the complex markings surrounding the comparatively reflective speculum (e.g. Streinzer *et al.*, 2010; Vigniolini et al., 2012) and, if successfully misled into alighting on the highly textured, three-dimensional labellum, various tactile cues come into play (e.g. Bradshaw *et al.*, 2010; Francisco and Ascensão, 2013). Pseudo-copulatory pollination has proven sufficiently unusual and intriguing to render the genus *Ophrys* something of a research industry in recent years, advancing the genus to the point when it can legitimately be described as a model system in reproductive biology studies (compare Kullenberg, 1961; Borg-Karlson, 1990; Paulus and Gack, 1990; Schiestl *et al.*, 1999; Mant *et al.*, 2005; Jersakova et al., 2006; Vereecken, 2009; Ayasse *et al.*, 2010; Schiestl and Johnson, 2013).

Despite this intense interest (or perhaps because of it?), *Ophrys* has also become a catalyst for occasionally intense debates, both evolutionary and taxonomic, that focus especially on the significance and fidelity of the plant–pollinator relationship (‘ethology’ *sensu*Bateman *et al.*, 2011) and the degree of relevance to the circumscription of species within the group (cf. Bateman *et al.*, 2011; Vereecken *et al.*, 2011; Bateman, 2012; Schlüter and Johnson, 2013; Paulus, 2015; Véla *et al.*, 2015; Cotrim *et al.*, 2016). However, in order to pursue well-informed discussions on these topics, it is first necessary to have at our disposal an equally well-founded phylogeny that reveals the evolutionary relationships of the major groups within the genus, and to use that phylogeny to infer the sequence in which the many putative adaptations of the *Ophrys* flower were acquired by each of those major clades.

The present literature includes at least two morphological phylogenetic studies (Devillers and Devillers-Terschuren, 1994; Francisco and Ascensão, 2015) and several molecular phylogenetic studies that either placed numerous members of the genus within a broader taxonomic context (Pridgeon *et al.*, 1997; Bateman *et al.*, 2003; Inda *et al.*, 2012; Tang *et al.*, 2015) or focused largely on *Ophrys* (Soliva *et al.*, 2001; Devey *et al.*, 2008; Breitkopf *et al.*, 2015). However, the results of such studies have proven to be both internally ambiguous and often strongly mutually contradictory. Here, we combine a newly assembled morphological cladistic analysis with a next-generation sequencing approach termed RAD-seq (restriction site-associated sequencing; e.g. Davey *et al.*, 2013; Eaton and Ree, 2013; MacCormack et al., 2013; Rubin et al., 2013; Wagner *et al.*, 2013; Massatti *et al.*, 2016; Olson *et al.*, 2016; Paun *et al.*, 2016). To date, next-generation sequencing studies of orchids have been few and have relied on sparse sampling across the entire family (e.g. Luo *et al.*, 2014; Deng *et al.*, 2015). Our objectives in the present study were (1) to determine the relationships among the major clades of bee orchids with considerably greater confidence and (2) to explore phenotypic evolution within the genus. We aimed not only to determine the sequence in which the many complex characters were acquired by various lineages but also to provide a credible reconstruction of the morphology of the most recent common ancestor of the genus.

## MATERIALS AND METHODS

### Plant materials

The authors were able to exploit extensive collections of silica gel samples of European orchids accumulated over a period of approximately 20 years in both London and Debrecen. Taxon selection was based primarily on the detailed molecular study of the genus by Devey *et al.* (2008), who identified six major groups using either concatenated plastid sequences or amplified fragment length polymorphism (AFLP) data, and ten groups (four of the ten being only subtly distinct from each other) using cloned nuclear ribosomal internal transcribed spacer (nrITS) sequences. Here, we selected a minimum of two microspecies from each of those ten groups, increasing sampling density in the two most molecularly and morphologically problematic groups [*sphegodes sensu lato* (*s.l.*) and *fuciflora s.l.*] to seven microspecies each, together constituting an ingroup of 32 samples (Table 1) that were distributed fairly evenly across (but not wholly coincident with) the 32 morphological groups circumscribed by Delforge (2006; later reduced to 29 groups by Delforge, 2016). A further two samples were selected as outgroups from among those genera shown by Bateman *et al.* (2003) to be closely related to *Ophrys*, specifically *Steveniella satyrioides* and *Serapias lingua*.

**Table 1.** Details of orchid samples subjected to RAD sequencing for the present study

Microspecies	Reference number	Locality	Delforge (2016) group	Devey *et al.* (2008) group
*Steveniella satyrioides*	RMB2327	Maçka, Turkey	[outgroup]	[outgroup]
*Serapias lingua*	RMB2621	Ghisonaccia, Corsica	[outgroup]	[outgroup]
*insectifera*	MVA-43259	Torri del Benaco, N Italy	*insectifera*	*insectifera* (A)
*insectifera*	MVA-43260	Muran, Slovakia	*insectifera*	*insectifera* (A)
*aymoninii*	RMB1235	Cauals, S France	*insectifera*	*insectifera* (A)
*neglecta*	RMB0651	Mattinata, S Italy	*tenthredinifera*	*tenthredinifera* (B)
*normanii*	RMB2023	Novusdomus, Sardinia	*tenthredinifera*	*tenthredinifera* (B)
*bombyliflora*	RMB2681	Sassari, Sardinia	bombyliflora*	*bombyliflora* (C)
*bombyliflora*	RMB1220	Gythio, Greece	bombyliflora*	*bombyliflora* (C)
*speculum*	RMB2030	Laconi, Sardinia	*speculum*	*speculum* (D)
*regis-ferdinandii*	RMB1098	Armakia, Chios, Aegean Gr.	*speculum*	*speculum* (D)
cf. *fusca*	RMB0665	Mattinata, S Italy	*fusca*	*fusca* (E)
*iricolor*	RMB1134	Olimbi, Chios, Aegean Gr.	*iricolor*	*fusca* (E)
*lutea*	RMB2084	Ferla, Sicily	*lutea*	*fusca* (E)
*apifera*	MVA-43261	Vila de Bispo, S Portugal	*apifera*	*apifera* (F)
*apifera*	SG-43262	Mekami, Albania	*apifera*	*apifera* (F)
*levantina*	RMB2207	Icel, S Turkey	*bornmuelleri*	*umbilicata* (J)
*umbilicata*	RMB2443	Kyalar, Cyprus	*umbilicata*	*umbilicata* (J)
*reinholdii*	RMB1166	Gythio, S Greece	*reinholdii*	*sphegodes* (G)
*argolica*	RMB1159	Mystras, S Greece	*argolica*	*sphegodes* (G)
*aveyronensis*	RMB2289	Guilhaumard, S France	*incubacea*	*sphegodes* (G)
*sphegodes* ^†^	SG-43263	Tatárszentgyörgy, Hungary	*sphegodes*	*sphegodes* (G)
*taygetica*	SG-43264	Taygeti, Greece	*mammosa*	*sphegodes* (G)
*lunulata*	MVA-37042	Ferla, Sicily	*lunulata*	*sphegodes* (G)
*benacensis*	MVA-37072	Gardone, N Italy	*bertolonii*	*sphegodes* (G)
*fuciflora*	MVA-43265	Borut, Croatia	*fuciflora* ^‡^	*fuciflora* (H)
*oxyrrhynchos* ^§^	MVA-43266	Palazzolo Acreide, Sicily	*fuciflora*	*fuciflora* (H)
*elatior*	RMB1945	Basel, Switzerland	*tetraloniae*	*fuciflora* (H)
*biancae*	MVA-43267	Ferla, Sicily	*bornmuelleri*	*fuciflora* (H)
*homeri*	MVA-43268	Lesvos, Aegean Greece	*heldreichii*	*scolopax* (I)
*oestrifera*	SG-43269	Budapest, Hungary	*oestrifera*	NA (?*scolopax*: I)^§¶^
*oestrifera*	SG-43270	Xizi, Azerbaijan	*oestrifera*	NA (?*scolopax*: I)^¶^
*scolopax*	MVA-37743	La Palme, S France	*scolopax*	*scolopax* (I)
*picta*	MVA-43271	Antequera, S Spain	scolopax	*scolopax* (I)

Collectors RB, R. Bateman (personal accession/image numbers); SG, Gábor Sramkó; AM, Attila Molnár (numbers indicate images and/or specimens deposited in the DE-Soo herbarium).

*Placed within the *tenthredifera* group by Delforge (2006).

^†^Not accepted as a Hungarian native by Delforge (2006).

^‡^Includes *O. holubyana* of Delforge (2006, 2016).

^§^Sample was preserved in ethanol rather than silica gel.

^¶^Unfortunately, a misidentified vegetative sample of *O. apifera* represented *oestrifera* in the tree of Devey *et al.* (2008).

### DNA extraction

Plant tissue samples were field collected in silica gel (or, in the case of *O. oxyrrhynchos*, 96 % ethanol) for later extraction of total genomic DNA. Between 1 and 30 mg of dried leaves and/or flowers were ground thoroughly in liquid nitrogen, then re-suspended in lysis buffer [2 % cetyltrimethylammonium bromide (CTAB), 20 mm EDTA pH 8, 100 mm Tris–HCl pH 9 and 1.4 mm NaCl]. After incubation at 65 °C for 60 min, the samples were centrifuged at 20 000 *g* for 10 min, then the supernatant was extracted with an equal volume of chloroform and centrifuged for 15 min at 20 000 *g*. The extraction procedure was repeated twice. DNA was precipitated with two volumes of 96 % ethanol and stored at –20 °C or below for 1 h. DNA was pelleted by centrifugation at 14 000 rpm for 30 min. The pellet was washed twice with 70 % ethanol, air-dried and re-dissolved in 40–100 μL of 0.1 m Tris (pH 7.5). The raw DNA extracts were then cleaned with the NucleoSpin gDNA clean-up kit (Macherey-Nagel) by following the manufacturer’s protocol. Finally, the double-stranded DNA content of each clean extract was measured fluorometrically by Qubit dsDNA HS Assay Kit on a Qubit v.3.0 fluorometer (ThermoFisher Scientific).

### Generation of RAD-seq data

The single-digest RAD-seq laboratory protocol was adapted from that detailed by Paun *et al.* (2016), with the following modifications. Given that the mean genome size of *Ophrys* approximates 1C = 10 pg (Leitch *et al.*, 2009; Bateman *et al.*, 2018), the RAD-seq library was prepared using the restriction enzyme *Sbf*I (New England Biolabs, Germany). For each individual we used 210 ng of starting plant material, shearing the restricted and P1-ligated DNA with a Bioruptor Pico (Diagenode) for two cycles of 30 s ‘on’, 60 s ‘off ‘. The fragments were constructed with a system of double inline barcoding: eight different 6 bp barcodes were inserted with the P1 adaptor in combination with five P2 barcodes, each 4 bp long. All barcodes differed by at least three sequence positions. The final libraries were submitted to VBCF Vienna (http://vbcf.ac.at/ngs) for paired-end 100 bp sequencing on two lanes of an Illumina HiSeq 2500 platform.

### Filtration of RAD-seq data

We optimized a bioinformatics pipeline for maximizing the recovery of loci across the coverage variation and phylogenetic depth present within our data, making use of the paired-end reads available. This was a dynamic process, and in the following account we present only the final analytical pipeline, optimized primarily on the number of variable sequence positions in the data and on the bootstrap support in the resulting trees. Given the vast number of parsimony-informative single nucleotide polymorphisms (SNPs) generated via RAD-seq, the bootstrap support and even the topologies resulting from various analytical approaches proved to be surprisingly sensitive to experimental parameters, most notably the permitted levels of missing individuals per site.

The raw Illumina paired-end reads were demultiplexed and quality filtered using the program ‘process_radtags’ from the suite STACKS v.1.35 (Catchen *et al.*, 2013), rescuing barcodes and RAD tags with a maximum of one mismatch. Next, the overlapping pairs of reads (i.e. originating from DNA fragments of a length smaller than twice the read length) were merged using FLASH v.1.2.11 (Magoc and Salzberg, 2011) under default settings. Only a subset of the pairs could be overlapped through this approach. We then employed the resulting contigs of variable length (i.e. between 96 and 180 bp) to build a catalogue of loci using pyRAD v.3.0.63 (Eaton and Ree, 2013). This software package allows for indels (expected in the phylogenetic framework of our study) and for unequal length of input sequences (resulting from overlapping read pairs) when clustering orthologous loci. The extended contigs were clustered with pyRAD using an 85 % similarity threshold to create RAD tags, by retaining only those loci with a minimum depth of coverage of six at each site and that were present in at least 60 % of the samples.

The longest individual contig was selected from each cluster for promotion to the final reference, which was encoded as a genome with as many ‘chromosomes’ as contigs. This construct has been further used to call variants based on all pairs of reads (i.e. overlapping or non-overlapping) generally following the Best Practices recommendations for DNA sequencing (DePristo *et al.*, 2011; Van der Auwera *et al.*, 2013) for Genome Analysis Toolkit (GATK) v.3 (McKenna *et al.*, 2010), but without marking and removing PCR duplicates. These are impossible to distinguish in RAD-seq data sets, given the consistent start of reads at the restriction cut site and, specific for our analysis, the frequent mapping stopping point at the 3’ end of the reference contigs.

After the reads were mapped with the MEM algorithm of BWA v.0.7.15-r1140 (Li and Durbin, 2009), the BAM files have been processes by sorting them by queryname and adding read groups with Picard tools v.2.1.0 (available from http://broadinstitute.github.io/picard). The IndelRealigner module from GATK v3.6-0-g89b7209 was used to improve local alignments around indels. Variants were further called for each sample in the GVCF mode of the GATK HaplotypeCaller to generate an intermediate gVCF. Next, we processed all samples in the cohort in a joint genotyping analysis with the GenotypeGVCFs module of GATK, employing the minimum phred-scaled confidence threshold of ten at which variants should be called. Thus, we followed GATK best practices recommendations for DNAseq. After retaining only SNPs with the SelectVariants module of GATK, the variants were further filtered out if any one of the following three criteria was fulfilled: (1) the quality normalized by the coverage (QD) was <2; (2) the Phred-scaled *P*-value for the Fisher’s exact test to detect strand bias (FS) was >60; or (3) the root mean square of mapping quality across all samples (MQ) was <40.

### Construction of unrooted and rooted trees

The resulting vcf file was further filtered using vcftools v.0.1.14 (Danacek et al., 2011), set to retain only those SNPs that are covered in at least 60 % of the individuals and show a minimum minor allele frequency of 0.065. Two vcf files were produced employing these settings: the first including all 34 individuals and the second omitting the two outgroup accessions (*Steveniella* and *Serapias*). The filtered vcf files were converted to phylip format by concatenating the SNP positions with PGDSpider v.2.0.8.2 (Lischer and Excoffier, 2012), summarizing heterozygosities as IUPAC ambiguities.

The ingroup-only file was further used to produce a phylogenetic network in SPLITSTREE v.4.3.11 (Huson and Bryant, 2006). Splits were created from uncorrected-p distances and visualized as a neighbour net within which each end node represents an individual accession.

A maximum likelihood (ML) phylogenetic tree with 1000 rapid bootstrap inferences, a GTR substitution matrix and GAMMA model of rate heterogeneity was inferred based on the all-individuals data set using RAxML v.8.2.9 (Stamatakis, 2014). The analysis was run using ascertainment bias correction (ASC), given that our data set contained only concatenated informative SNP positions. Prior to the analysis, RAxML demanded the removal of 90 ‘invariable’ sites from the data set that represented exclusively heterozygote polymorphism. The RAxML results were graphically visualized and edited in FigTree v.1.4.2 (available from http://tree.bio.ed.ac.uk/software/figtree/). The same matrix was subsequently subjected to Bayesian tree-building using MrBayes (analytical details are provided in the legend to Supplementary Data Fig. S1).

### Morphological cladistics

Entities chosen for morphological cladistic analyses were informed primarily by the cloned ITS analysis of Devey *et al.* (2008), who tentatively recognized ten macrospecies (labelled A–J). Here, two unusually morphologically diverse molecular macrospecies, *umbilicata* and *sphegodes*, were further divided into two and three subgroups respectively, in order to limit the already considerable number of matrix cells coded as polymorphic. This decision increased the number of ingroup taxa to 13. The three selected outgroups are the earliest divergent members of the three clades that have been shown through DNA data to be closely related to, and diverge immediately prior to, *Ophrys*, namely *Steveniella*, *Neotinea* and *Orchis* (Bateman *et al.*, 2003; Tang *et al.*, 2015).

Morphological data were derived from numerous sources and were input into MacClade v4.05 (Maddison and Maddison, 2002). When compiling our character list (Table 2), characters were, where feasible, derived from the literature and supplemented with original observations by the present authors. The only previous genus-wide morphological cladistic analysis of *Ophrys* (Devillers and Devillers-Terschuren, 1994), plus a more focused analysis of a major clade within the genus (Francisco *et al.*, 2015), provided a useful foundation for macromorphological characters. The detailed study by Bradshaw *et al.* (2010), as slightly amended by Francisco and Ascensão (2013), informed our choice of micromorphological characters. We deliberately adopted a more conservative approach to scoring micromorphological characters than did Francisco *et al.* (2015), suspecting that epidermal features are prone to extensive pleiotropy; in addition, their potentially valuable empirical observations on the size and location of osmophores would need to be extended across the genus in order to qualify for inclusion in the matrix. Also tangentially helpful were the well-known European orchid flora by Delforge (2006, 2016), the more technical treatise on flower morphology and function by Claessens and Kleynen (2011), and the monograph of the European *Ophrys* by Pedersen and Faurholdt (2007). More focused scanning electron microscopy (SEM)-based studies of ovule testae (Galán Cela *et al.*, 2014) and pollen exines (Barone Lumaga *et al.*, 2006) failed to yield usable characters and, surprisingly, comparative studies of pollinaria have not to our knowledge been attempted within the genus (e.g. we were tempted to score as distinct the slender caudicles of *O. apifera* that are considered responsible for its facultative autogamy, but we lacked the necessary comparative data).

**Table 2.** Characters scored for morphological cladistic analysis of nine major groups and four further subgroups within the genus Ophrys

*A.*	*Labellum: shape*
1.	Outline of flattened labellum longer than broad (0): broader than long (1).
2.	Lateral sinuses well-developed (0): absent or poorly developed (1)
3.	Lateral lobes indistinct, labellum more or less flat (0): project forward slightly beyond speculum (1): project forward well beyond speculum (2).
4.	Mid-lobe more or less flat (0): weakly convex (1): strongly convex (2).
5.	Mid-lobe sinus deep (0): shallow or absent (1).
6.	Mid-lobe apex projects downward (0): strongly recurved (1).
7.	Outer half of labellum more or less occupying the same plane as the upper half (0): clearly bent forward when viewed laterally (1).
8.	Base of labellum more or less flat (0): possessing V-shaped groove or geniculate ‘step’ (1).
*B.*	*Labellum: appendages*
9.	Labellar spur present (albeit short or vestigial) (0): absent (1).
10.	Appendix absent (0): subdued, resembles rest of labellum (1): discrete structure, bright yellow (2).
11.	Epidermis wholly papillose (0): at least partly pilose (i.e. trichomes present) (1).
12.	Ciliae (coarse trichomes) absent (0): diffuse and comparatively homogeneous (1): more prominent on labellar ‘shoulders’ (2): prominent along entire labellar margin (3): confined to basal ‘throat’ (4).
*C.*	*Labellum: speculum*
13.	Speculum location, absent (0): immediately adjacent to stigma (1): connected to stigma by pale strips (2): isolated from stigma (3).
14.	Speculum shape, absent (0): solid or U or W (1): oo or II (2): H (3): complex ring, often also bearing outward projections (4).
15.	Speculum marginal pale zone absent (0): indistinct (1): distinct (2).
16.	Longitudinal bosses and intervening papillose groove absent (0): present (1).
17.	Epidermal cells papillose (0): pilose (1): flat (2).
*D.*	*Stigmatic region*
18.	Basal field absent (0): within (inner) labia or temporal calli (1): outside labia or below temporal calli (2).
19.	Stigmatic cavity cordate (0): spherical (1): hemispherical (2).
20.	Floor of stigmatic cavity smooth (0): pilose (1).
21.	Marginal (inner) labia of stigmatic cavity absent (0): present (1).
22.	Temporal calli (pseudoeyes) absent (0): present (1).
23.	Staminodial calli absent or obscure (0): well-developed (1).
*E.*	*Gynostemium*
24.	Gynostemium short with blunt apex (0): elongate with acute apex (beaked connective) (1).
25.	Bursicle entire (0): completely divided into two segments (1).
*F.*	*Lateral petals*
26.	Petals 75–100 % of median sepal length (0): 50–75 % of median sepal length (1): <50 % of median sepal length (typically triangular) (2).
27.	More or less planar (0): lateral margins enrolled backward (1): strongly apically reflexed (2).
28.	Epidermal cells glabrous (0): pilose (1).
29.	Ciliate margins absent (0): clearly present (1).
*G.*	*Sepals*
30.	Thickened margin absent (0): present (1).
31.	Median sepal posture directed forward, forming hood with connivent lateral petals (0): directed forward but not forming hood (1): erect (2).
32.	Median sepal shape broadly lanceolate/ovate (0): obovate (1).
33.	Median sepal has width <70 % length (0): width >70 % length (1).
34.	Lateral sepal base colour yellow-green/green (0): pink/purplish-brown (1).
35.	Dark staining of lower half of sepal absent (0): present (1).
*H.*	*Stem and inflorescence*
36.	Orientation of labellum determined via 180° torsion of pedicel and/or ovary (0): inversion of bud (1).
37.	Bracts more or less membranous (0): foliose (1).
38.	Inflorescence dense with numerous small flowers (0): lax with few large flowers (1).
*I.*	*Leaves*
39.	Leaf number and position more or less concentrated in basal rosette, ovate–lanceolate (0): distributed along lower part of stem, narrowly lanceolate (1): basal, reduced in number to one or two (2).
40.	Leaf and stem purple anthocyanins absent (0): suffused (1): discrete dashes (2).
*J.*	*Tubers and roots*
41.	Stolons absent (0): present (i.e. forms clonal colonies) (1).
*K.*	*Cytology*
42.	*n* = 21/?20 (0): 18/?19 (1).
43.	2*n* = 2*x* (0): 2*n* = 3*x* and/or 4*x* (1).

## RESULTS

### RAD-seq network and tree

The average number of raw pairs of reads per accession retained after demultiplexing and quality filtering was 1.35 million (s.d. 0.50 million). The single ethanol-preserved sample, which had initially given us concern, in practice performed as well as the average silica gel-preserved sample. The reads had a length of 94 bp forwards and 96 bp for the pair-reads after removing the barcode sequence. In total, 8.1 million pairs (17.6 % of the total) could be successfully extended with FLASH up to a length of 180 bp. After clustering using PyRAD, a total of 127 983 bp over 751 contigs were retained as a pseudo-reference for calling variants. The raw variant file contained 16 758 SNPs and 3022 indels. After filtering with the settings specified above, we retained 4159 SNPs (of which 164 were tri-allelic across all samples) for the 34-individual data set and 4060 SNPs for the ingroup-only data set. This filtration process yielded an average across the data set of one SNP retained every 30.8 bp. Relative to variable positions only, 5 % of the SNPs were represented by a heterozygote call, whereas across the entire reference and individuals, 0.2 % of positions were heterozygote. The raw data have been deposited in the NCBI Short Reads Archive (BioProject ID PRJNA400088, SRA Study ID SRP116164) and the processed matrix in the Dryad Digital Respository (doi: http://dx.doi.org/10.5061/dryad.s420s); this matrix was used as the basis of all subsequent tree-building protocols.

The unrooted SplitsTree network depiction of the data (Fig. 2) bears considerable resemblance to previously published molecular trees of the genus *Ophrys*. Three main, comparatively highly disparate lineages are evident: (1) *insectifera*; (2) a poorly resolved plexus containing *speculum* plus *bombyliflora* plus *tenthredinfera* plus *fusca* (the latter occupying an unusually long branch and sister to *tenthredinifera*); and (3) *apifera* plus *umbilicata* plus a much less well-resolved mélange that encompasses members of the *sphegodes*, *fuciflora* and *scolopax* groups, each sample occupying a much shorter branch. When that mélange is considerably magnified (Fig. 2 inset), it becomes evident that of these three ITS-based groups circumscribed by Devey *et al.* (2008), only the *sphegodes* group is cohesive. The topology nests the *fuciflora* group (H: *fuciflora*, *elatior*, *biancae* and *oxyrrhynchos*) within the *scolopax* group (I), separating the western *scolopax* group members (*scolopax* plus *picta*) from the eastern members (*homeri* plus *oestrifera*). However, the tree provides tentative evidence that the western *scolopax* plus *picta* could form the basis of a molecularly distinguishable group (Fig. 2 inset).

**Fig. 1.**
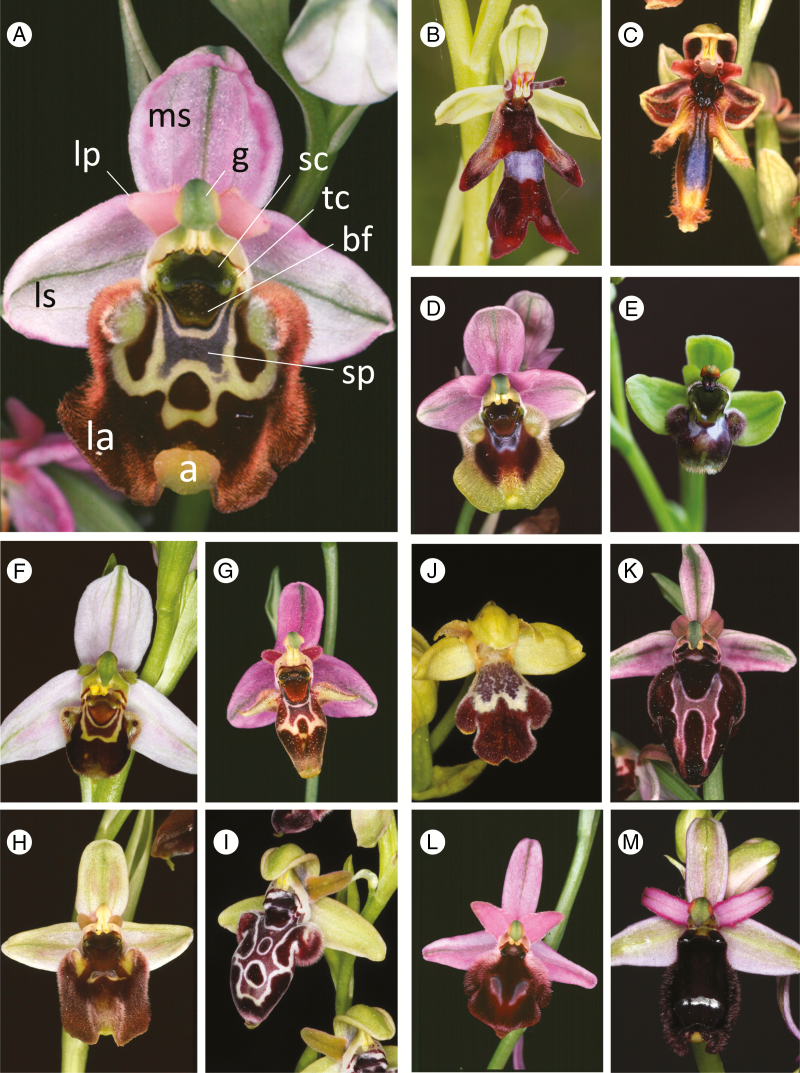
Flowers of 13 microspecies representing the nine molecularly circumscribed macrospecies (groups) of *Ophrys* discussed in the present phylogenetic study, together with four further subgroups created for specific use in our morphological cladistic analysis. (A) *O. episcopalis*, Crete (*fuciflora* group, *fuciflora* subgroup: H’1), (B) *O. insectifera*, UK (*insectifera* group: A), (C) *O. regis-ferdinandii*, Chios (*speculum group*: D), (D) *O. grandiflora*, Sicily (*tenthredinifera* group: B), (E) *O. bombyliflora*, Sardinia (*bombyliflora* group: C), (F) *O. apifera*, Sicily (*apifera* group: F), (G) *O. oestrifera*, Chios (*fuciflora* group, *scolopax* subgroup: H’2), (H) *O. bornmuelleri*, Cyprus (*umbilicata* group, *bornmuelleri* subgroup: J2), (I) *O. kotschyi*, Cyprus (*umbilicata* group, *umbilicata* subgroup: J1), (J) *O. israelitica*, Cyprus (*fusca* group), (K) *O. spruneri*, Crete (*sphegodes* group, *sphegodes* subgroup: G2), (L) *O. argolica*, Peloponnese (*sphegodes* group, *argolica* subgroup: G1), (M) *O. bertolonii*, Sicily (*sphegodes* group, *bertolonii* subgroup: G3). Labels on (A): la, labellum (lip); lp, lateral petal; ms, median sepal; ls, lateral sepal; g, gynostemium (column); sc, stigmatic cavity; tc, temporal callosity (pseudoeye); bf, basal field; sp, speculum; a, appendix.

**Fig. 2.**
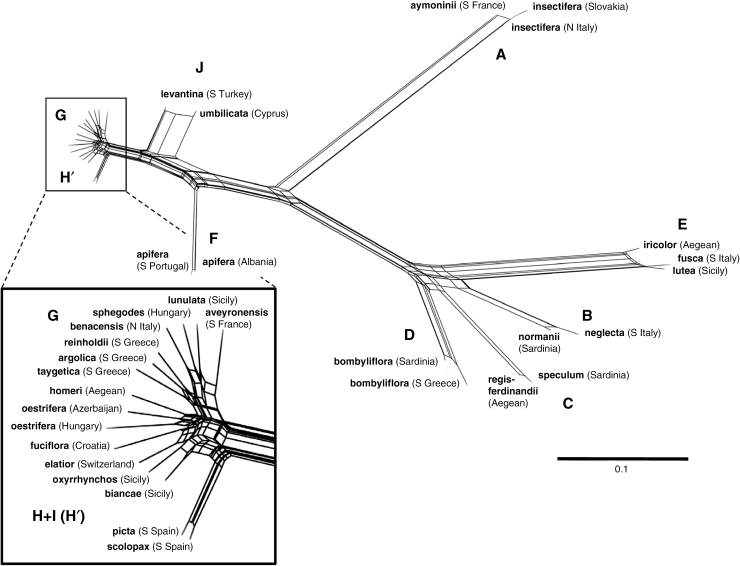
Unrooted SplitsTree network based on 4060 RAD-seq-derived SNPs for 32 plants that together represent the ten putative *Ophrys* macrospecies (A–J) illustrated in Fig. 1. Inset: magnified view of topology for representatives of groups G–I. Details of samples are given in Table 1.

**Fig. 3.**
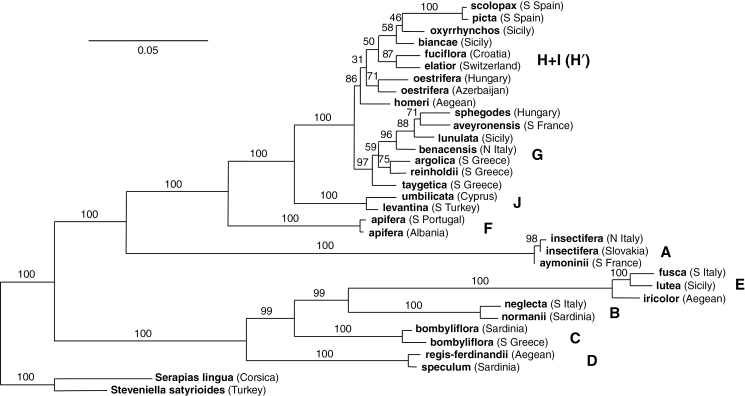
Rooted RAxML tree of RAD-seq data for the same 32 plants that formed the basis of Fig. 2, plus two outgroup accessions. The tree is based on 4159 high-quality, filtered SNPs. Values above the branch are bootstrap values, and groups A–J of Devey *et al.* (2008) are labelled. Details of samples are given in Table 1.

In the rooted ML phylogenetic tree derived from our finalized RAD-seq matrix (Fig. 3), eight of the ten ITS clades recognized by Devey *et al.* (2008) received at least 99 % bootstrap support, falling slightly to 97 % when estimating the cohesion of the *sphegodes* group. However, the RAxML topology follows the SplitsTree topology in nesting the *fuciflora* group (H) of Devey *et al*. within the *scolopax* group (I) and, in addition, shows *scolopax* plus *picta* as being nested within the *fuciflora* group. Admittedly, branches in this region of the tree attract only weak bootstrap support values ranging from 31 to 56 %, and even the combined H + I group (here designated H’) is less well supported (bootstrap support 86 %) than are groups A–G. Bayesian analysis performed on the same underlying matrix (Supplementary Data Fig. S1) yielded an identical topology, strikingly similar relative branch lengths, and posterior probability values that indicated points of least statistical certainty among microspecies in the same two locations: the branches immediately above *homeri* and *taygetica*, respectively.

By rejecting monophyly of group I, these results reduce the number of molecular clades recognized by us to nine from the initial figure of ten tentatively established by Devey *et al.* (2008). Moreover, most relationships within the better sampled but especially problematic *sphegodes* and combined *fuciflora–scolopax* groups received negligible statistical support in the rooted tree and lack cohesion in the unrooted network irrespective of the tree-building algorithm used. They clearly cannot be relied upon, as they show levels of molecular divergence no greater than those observed between two of the four pairs of samples that represent the same two microspecies: *O. bombyliflora* and *O. oestrifera*.

Our main interest in the resulting network (Fig. 2) and tree (Fig. 3) was exploring relationships among the ten (now apparently nine) molecularly delimited macrospecies. This is far from the first study to do so, but here we have the advantage of accessing a far larger, genome-wide data set than those made available in previous studies. We compared the present RAxML topology and branch lengths with both those generated in previous studies and the morphological topology generated during the present study, before mapping the morphological characters across the molecular topology to explore evolutionary pattern and process.

### Comparison of the RAD-seq topologies with those of previous molecular studies of *Ophrys*

**Fig. 4.**
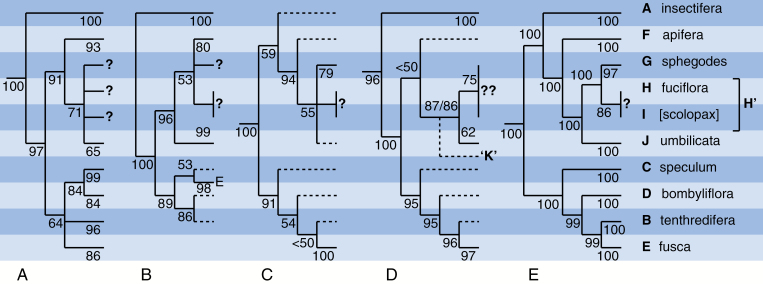
Comparison of topologies obtained in previous phylogenetic studies of *Ophrys*, reduced to the ten macrospecies (labelled A–J) recognized by Devey *et al.* (2008). (A) Devey *et al.*, 2008, fig. 2; ITS, MP. (B) Devey *et al.*, 2008, fig. 2; three plastid regions, MP. (C) Soliva and Widmer, 2001, fig. 1; ITS + one plastid region, MP. (D) Breitkopf *et al.*, 2015, fig. 1; six low-copy nuclear genes, ML. (E) Present study; RAD-seq, ML. MP, maximum parsimony; ML, maximum likelihood. Numbers associated with branches are bootstrap values. Dashed branches represent only a single analysed sample and so do not test monophyly of the relevant macrospecies. It was necessary to shift the horizontal position of the *fusca* group, E, in (B), and to interpolate a putative additional group based on *O. heldreichii*, K, in (D). In (B), the basal position of the *insectifera* group, A, was dictated by its use as the *de facto* outgroup. Question marks denote indistinguishable groups.

The present RAD-seq tree is compared in Fig. 4 with four previous DNA-based trees that collectively were generated from a wide range of genic regions and reflected widely varying numbers of analysed samples (cf. Soliva *et al.*, 2001; Devey *et al.*, 2008; Breitkopf *et al.*, 2015). Unsurprisingly, our RAD-seq tree (Fig. 4E) confirms the monophyly of the genus *Ophrys* that was evident in all previous molecular and morphological studies.

None of the five summarized studies was able to differentiate confidently as two monophyletic entities the *fuciflora* (H) and *scolopax* (I) groups tentatively established by Devey *et al.* (2008), making it clear that these groups should indeed be unified into a single group of *fuciflora sensu latissimo* (H’). The *sphegodes* group also gained only tentative circumscription in the plastid tree of Devey *et al.* (2008). More notably, the low-copy nuclear gene tree of Breitkopf *et al.* (2015) was unable to distinguish conclusively among all four of the groups (G–J) placed distal to *apifera*, instead intimately intermingling members of the *fuciflora–scolopax* group with those of the *sphegodes* group.

Moving on to consider the topology determined for the nine *bona fide* macrospecies, all five trees are consistent with Bateman’s long-standing assertion that three subgenera should be recognized, based on groups A, B–E and F–J, respectively. Three of the five trees shown in Fig. 4 place *insectifera* (A) as the earliest divergent lineage in the genus (though in tree 4B this placement simply reflects the use of *insectifera* as outgroup). Only in the present RAD-seq tree (Fig. 4E) and – with much lower bootstrap support – in the tree of Soliva *et al.* (2001) (Fig. 4C) was *insectifera* placed as sister to the *apifera*–*umbilicata* clade (F–J).

All five trees concur, most with strong statistical support, that groups B–E form a clade, albeit a clade that is exceptionally morphologically heterogeneous. Moreover, three of the five trees agree (again in most cases with strong statistical support) that the *speculum* group was first to diverge, followed by the *bombyliflora* group, thus leaving the *tenthredinifera* and *fusca* groups as a morphologically disparate sister-pair. Only the trees of Devey *et al.* (2008) contradict this topology; their cloned ITS tree paired *bombyliflora* with *speculum* whereas their plastid tree paired *bombyliflora* with *tenthredinifera*, in both cases with at least 80 % bootstrap support.

All five trees also agree that groups F–J form a clade, though with negligible bootstrap support in the case of Breitkopf *et al.* (2015). Most studies place the *apifera* group as earliest divergent within this clade, the exception being the plastid tree of Devey *et al.* (2008), which placed *apifera* in a more derived position. Above this point, most studies failed to resolve confidently the three remaining groups: *umbilicata* (J), *sphegodes* (G) and *fuciflora–scolopax* (H’). The present RAD-seq tree usefully confirms the earlier suggestion of Devey *et al.* (2008) that *umbilicata* was the earliest to diverge among groups J, G and H’, leaving *sphegodes* and *fuciflora–scolopax* as a derived sister-pair. This is also the first study to resolve as mutually monophyletic both members of this pair with strong statistical support.

The most surprising aspect of the tree of Breitkopf *et al.* (2015) was the placement of the eastern Mediterranean microspecies *O. heldreichii* below the *umbilicata* group (Fig. 4D); they argued that it represented a potentially novel macrospecies, which they labelled K. However, morphologically, this microspecies is clearly a member of the *fuciflora–scolopax* group (H’), a placement that was supported by the ITS tree of Devey *et al.* (2008). Unfortunately, *O. heldreichii* was not included in the present study, though we did analyse *O. homeri*, a microspecies that was assigned on morphological grounds to his *heldreichii* group by Delforge (2006, 2016). In our tree, *O. homeri* occurs with 86 % bootstrap support in clade H’, residing alongside morphologically similar microspecies, rather than being placed immediately above the *apifera* group as would be predicted by the tree of Breitkopf *et al.* (2015) (Fig. 4).

Given that RAD-seq draws its characters from across the entire genome, our RAD-seq tree also merits comparison with the results of the similarly genome-wide – if less technologically sophisticated – AFLP ordination of Devey *et al.* (2008). Unlike RAD-seq, their AFLP ordination was unable to distinguish between members of the *sphegodes* and *fuciflora–scolopax* groups, but, more surprisingly, AFLP data also failed to distinguish clearly among the *speculum*, *bombyliflora* and *tenthredinifera* groups (B–D), thereby contradicting – or, more accurately, failing to support fully – all five of the phylogenies summarized in Fig. 4.

Although Fig. 4 does not present proportional phylogenetic branch lengths, comparative branch lengths within trees nonetheless merit brief discussion. Most notably, the low-copy nuclear gene tree of Breitkopf *et al.* (2015) implied a mutation rate in the B–E clade that is approximately twice that inferred for the remainder of the genus. One of us (R.B.) previously suspected that this inference actually reflected misrooting of their tree. However, the present RAD-seq tree (Fig. 3) partly supports the tree of Breitkopf *et al*., in that it maintains the previous impression of a modestly elevated mutation rate. Nonetheless, in our tree, this assertion of rate acceleration applies only to the *fusca* group (E), perhaps helping to explain the large genetic distance observed in AFLP data by Devey *et al.* (2008) that separated the *fusca* group (E) from the remaining members of the B–E clade.

### Morphological cladograms

In total, 43 characters (Table 2) were eventually scored for the 16 selected taxa, yielding a matrix of 688 cells (Table 3); 29 of the characters were bistate and the remainder were multistate, yielding a potential maximum number of 62 apomorphic states available to resolve the 16 taxa. *Ophrys* species are remarkably homogeneous vegetatively, so viable vegetative characters were few and none proved to be phylogenetically informative within the ingroup. The sepals and lateral petals added ten characters and the gynostemium plus stigma a further nine characters. Inevitably, the complex labellum contributed the largest number of characters (17). Most characters could be coded for most taxa; only 0.9 % of the matrix cells were scored as unknown, the uncertainty being largely restricted to chromosome numbers within *Ophrys*. However, as many as 8.1 % of the cells were scored as polymorphic, the polymorphism being distributed across 15 of the 43 characters. The worst affected character was lateral sepal colour (C34), where nine of 16 cells were eventually scored as polymorphic; the most polymorphic single cell affected the *O. reinholdii* subgroup, whose members collectively presented four of the five character states developed by us to summarize that most complex of features, speculum shape (C14).

**Table 3.** Morphological cladistic matrix for nine major groups and four additional subgroups within the genus *Ophrys*

Taxon	1	2	3	4	5	6	7	8	9	10	11	12	13	14	15	16	17	18	19	20	21	22	23	24	25	26	27	28	29	30	31	32	33	34	35	36	37	38	39	40	41	42	43
*Orchis anthropophora*	0	0	0	0	0	0	0	0	0	0	0	0	0	0	0	0	0	0	0	0	0	0	0	0	0	0	0	0	0	0	0	0	0	0	0	0	0	0	0	0	0	0	0
*Neotinea maculata*	0	0	0	0	0	0	0	0	0	0	0	0	0	0	0	0	0	0	0	0	0	0	0	0	0	0	0	0	0	0	0	0	0	*0&*1	0	0	0	0	0	2	0	0	0
*Steveniella satyrioides*	0	0	0	0	1	0	0	0	0	0	0	0	0	0	0	0	0	0	1	0	0	0	0	0	0	0	0	0	0	0	0	0	0	1	0	0	0	0	2	1	0	1	0
*fusca*	0	0	0	1	1	0	0*&1*	1	1	0	1	*1&*4	1	1	*0&*1*&2*	0	1	0	1	1	0	0	0	0	1	*0&*1	0	1	0	1	1	*0&*1	0	0*&1*	0	1	1	1	0	0	0	1	0*&1*
*insectifera*	0	0	0	1	1	0	0	0	1	0	1	0	3	1	0	0	1	1	2	0	1	1	0	0	1	1*&2*	1	1	0	1	1	0	0	0	0	1	1	1	1	0	0	1	0
*speculum*	0	0	0	1	1	0	0	0	1	0	1	3	1	1	*1&*2	1	2	1	2	0	1	1	1	0	1	2	2	1	1	1	1	0	0	0	1	1	1	1	0	0	0	1	0
*bombyliflora*	1	0	1	2	1	1	0	0	1	1	1	2	2	1	1	0	0	2	2	0	1	1	1	0	1	2	0	1	0	1	1	*0&*1	1	0	0	1	1	1	0	0	1	1	0
*tenthredinifera*	1	1	1	*1&*2	1	0	0	0	1	2	1	1	2	1	*1&*2	0	2	2	2	0	0	1	0	0	1	2	0	1	0	1	2	*0*&1	1	1	0	1	1	1	0	0	0	1	0*&1*
*apifera*	1	0	1	2	1	1	0	0	1	1	1	2	2	1	2	0	1	2	2	0	0	1	0	1	1	2	1	1	0	1	2	0	0	1	0	1	1	1	0	0	0	1	0
*umbilicata* (*umbilicata* 1)	1	1	1	2	1	0	0	0	1	2	1	2	2	4	2	0	0*&1*	2	2	0	0	1	0	1	1	1*&2*	0	1	0	1	2	*0&*1	0	0*&1*	0	1	1	1	0	0	0	?	?
*bornmuelleri* (*umbilicata* 2)	1	0	1	2	1	0	0	0	1	2	1	1	2	*2&*3	*1&*2	0	1	2	2	0	0	1	0	1	1	2	0	1	0	1	1	*0&*1	0	0*&1*	0	1	1	1	0	0	0	?	?
*fuciflora* (*fuciflora* 1)	1	1	1*&2*	2	1	0	0	0	1	2	1	1*&2*	2	*3&*4	2	0	1	2	2	0	0	1	0	1	1	*1&*2	0	1	0	1	2	0	0	*0&*1	0	1	1	1	0	0	0	1	0*&1*
*scolopax (fuciflora* 2)	1	0	*1&*2	2	1	0	0	0	1	2	1	2	2	4	2	0	1	2	2	0	0	1	0	1	1	1*&2*	0	1	0	1	2	0	0	*0&*1	0	1	1	1	0	0	0	1	0
*reinholdii* (*sphegodes* 1)	1	1	*0&*1	*1&*2	1	0	0	0	1	1	1	*0&1&*2	2*&3*	*2&*3*&4*	1*&2*	0	1	2	2	0	0	1	*0&*1	1	1	1	0	1	0	0	2	0	0	0*&1*	0*&1*	1	1	1	0	0	0	?	?
*mammosa* (*sphegodes* 2)	1	1	*0&*1	1*&2*	1	0	0	0	1	1	1	1*&2*	2*&3*	*1&2&*3*&4*	1*&2*	0	1	2	2	0	0	1	0	1	1	1	0	1	0	0	2	0	0	0*&1*	0*&1*	1	1	1	0	0	0	1	0
*bertolonii* (*sphegodes* 3)	*0&*1	1	0*&1*	2	1	0	*0&*1	0	1	1	1	1	*2&*3	1*&2&3*	1*&2*	0	2	1	2	0	0	1	0	1	1	1	0	1	0	0	2	0	0	*0&*1	0	1	1	1	0	0	0	1	0

In the case of polymorphic cells, the character states considered less frequent across the range of microspecies comprising that group are denoted in italics.

**Fig. 5.**
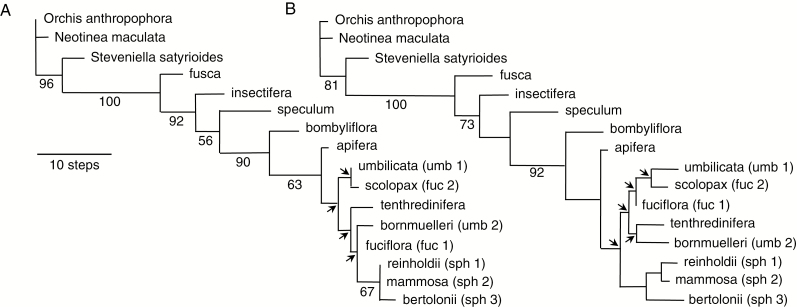
Morphological cladograms generated via maximum parsimony from a matrix of 13 ingroup plus three outgroup species. (A) One of the nine most-parsimonious trees generated from the initial matrix that included polymorphic cells. (B) One of three most-parsimonious trees generated from the present morphological cladistic matrix after all polymorphic cells had been resolved in favour of the most frequent character state within each. Arrowed nodes collapsed in the respective strict consensus trees. Branch lengths reflect Acctran optimization. Numbers on branches are bootstrap support values.

Data were transferred as nexus files from ‘MacClade’ v4.05 (Maddison and Maddison, 2002) to PAUP v4.0b10 (Swofford, 2001) in order to generate maximum parsimony trees, employing branch-and-bound searches. When the matrix was analysed in its original form, rich in polymorphic cells, three of the characters were revealed as constant and a further nine as parsimony uninformative. Under amb– branch collapse criteria, the matrix generated nine most-parsimonious trees of length 74 steps, consistency index 0.770 (0.734 without autapomorphies) and retention index 0.840. A representative example of the nine trees is shown as Fig. 5A.

Because it was immediately clear that the polymorphic cells were introducing an undesirable level of ambiguity into the analysis, an unambiguous version of the matrix was then created by reducing each polymorphic cell to the most frequent of the states observed within that coded taxon. When implementing this data simplification protocol we recognized that we were unable to determine whether, within each taxonomic group, the most frequent state of the polymorphic character was more likely to be plesiomorphic or apomorphic. This revised, non-polymorphic matrix deemed only one character to be constant and a further nine characters to be parsimony uninformative. This approach reduced the number of most-parsimonious trees to only three though, predictably, the removal of the polymorphic cells considerably increased both tree length and perceived homoplasy levels; the resulting trees were of length 95 steps, consistency index 0.642 (0.595 without autapomorphies) and retention index 0.732 (Fig. 5B).

Statistical support values for both matrices were estimated in PAUP through 1000 bootstrap replicates. Average bootstrap values were slightly lower for the non-polymorphic matrix. Despite the comparatively small number of most-parsimonious trees generated in each analysis, in both cases five of the more distal nodes collapsed in the strict consensus tree (indicated with arrows in Fig. 5). This instability primarily reflected uncertainty in the placements of the *apifera* group and the *bornmuelleri* subgroup of the *umbilicata* group, though it becomes difficult to draw any conclusions from this portion of the trees other than monophyly of the *sphegodes* group (G).

### Comparison of the morphological cladistic topologies with the molecularly delimited groups

The morphological cladistic tree set (Fig. 5) resembles the RAD-seq ML tree (Fig. 3) more closely in branch lengths than in topology. In both cases, the longest branch separates the outgroup from the ingroup and, within the ingroup, the lower branches are much longer, and hence more statistically robust, than those upper branches subtending groups F–J. All nodes subtending groups F, G, H’ and J collapse in the strict consensus trees for morphology, irrespective of the presence or absence of polymorphic cells (compare Fig. 5A vs. B). Groups J, H’ and G were represented in the morphological matrix by two, two and three morphological subgroups respectively, but only the *sphegodes* group (G) survived cladistic analysis intact, receiving approximately 80 % bootstrap support. As we anticipated, *scolopax* of group H’ was drawn toward *umbilicata* of group J, whereas *fuciflora* of group H’ was drawn toward *bornmuelleri* of group J, this second morphological pairing having labella that are less three-dimensional and less boldly marked than the first pairing.

Most notable is the presence within this less well-resolved F–J clade of *tenthredinifera* (group B). In the RAD-seq tree, *tenthredinifera* is seen as a derived member of the B–E clade, but in the morphological trees it is placed close to *fuciflora* and *bornmuelleri*, reflecting several morphological similarities. Beneath the comparatively poorly resolved F–J clade, the remaining groups form a more statistically robust progression that places *insectifera* (group A) within the three remaining members of the B–E clade. *Speculum* (C) and *bombyliflora* (D) diverge above *insectifera*, whereas the *fusca* group (E) is shown as being the earliest to diverge within the genus. The resulting topology is broadly similar to that generated during the only previous genus-wide morphological cladistic analysis of *Ophrys* (Devillers and Devillers-Terschuren, 1994), which provided some foundations of the present morphological analysis.

Equivalent Neighbor–Joining (NJ) trees (not shown) enhanced the perceived divergence between outgroups and ingroup. They broadly resembled the equivalent maximum parsimony trees in both topology and branch lengths, but the *speculum* group became paired with the *insectifera* group. The polymorphic NJ tree placed the *tenthredinifera* group immediately above the *bombyliflora* group, whereas in the non-polymorphic tree it was twinned with the *fuciflora* subgroup and hence was placed higher in the tree. In both cases, the *sphegodes* group was shown as monophyletic and earlier divergent than the various members of groups J and H’. Only in the non-polymorphic NJ tree were the two subgroups of the *umbilicata* group (J) paired together; also, this tree featured much longer terminal branches than those calculated for the more ambiguous polymorphic matrix.

To the best of our knowledge, the only other relevant morphological matrix is that compiled by Francisco *et al.* (2015), who confined their attention to the groups that together constituted the previously molecularly delimited B–E clade. They subjected these four groups to a detailed and meticulous morphological cladistic analysis, tacitly assuming their collective monophyly by employing only a single outgroup – the phylogenetically derived, apomorphy-rich (and here discredited) *scolopax* group. Their matrix of 45 characters was meticulously compiled and analysed, but relied heavily on numerous micromorphological characters describing details of the labellar epidermis that are highly likely to suffer from extensive pleiotropy, and thus fail the prior requirement for character independence. Francisco *et al.* (2015) favoured three of the 15 fully resolved topologies that theory dictates can potentially be derived from a four-taxon statement such as theirs, but none of their preferred topologies was obtained from either our RAD-seq or our morphological matrices. Most notably, their matrix yielded the *fusca* group as the earliest diverging of the four lineages under maximum parsimony but as part of the latest-diverging lineage under Bayesian analysis, thereby simultaneously resembling – in this feature at least – both our morphological tree and our RAD-seq tree, respectively (compare Figs 3 and 5). Francisco *et al.* (2015) similarly found the *tenthredinifera* group to behave as a destabilizing ‘wildcard’ taxon *sensu*Nixon and Wheeler (1992) – an understandable outcome when viewed in the light of our own results.

### Mapping of the morphological cladistic character states across the RAD-seq tree

When most-parsimonious trees resulting from the two morphological matrices (Fig. 5) were constrained to the topology generated by RAxML analysis of the RAD-seq SNP matrix (Fig. 3), trees derived from the polymorphic matrix increased in length by 19 steps and those derived from the non-polymorphic matrix increased by 20 steps (i.e. +26 % with polymorphic coding, +21% with unified coding). Unsurprisingly, perceived homoplasy increased substantially in both matrices. In the case of the non-polymorphic matrix, the consistency index decreased to 0.606 (0.561 excluding uninformative characters) and the retention index fell to 0.624; corresponding figures for the non-polymorphic matrix were 0.530 (0.481) and 0.575.

**Fig. 6.**
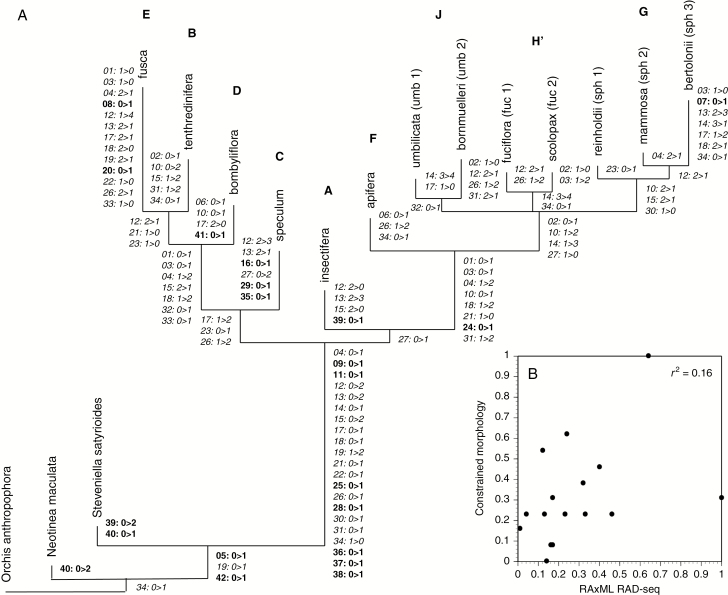
(A) Integrated phylogeny generated by constraining the 43 characters (33 informative) of the non-polymorphic morphological matrix to the topology dictated by the RAD-seq tree illustrated in Fig. 3. Character numbers and states reflect those given in Appendix 1. Acctran optimization; non-homoplastic character-state transitions shown in bold, homoplastic character-state transitions shown in italics. (B) Plot of standardized branch lengths for molecularly constrained morphology against the RAxML RAD-seq tree; both sets of branch lengths have been standardized to unit variance.

Imposing the molecular backbone constraint allowed us to map the morphological character states across the molecular topology (Fig. 6). Our objectives were to identify those characters most prone to homoplasy and to reconstruct the hypothetical morphology of shared ancestors, not least that occupying the basal node of the tree as the common ancestor of all extant lineages within the genus. It was then feasible to standardize the lengths of branches within the ingroup to unit variance relative to the longest branch, thereby permitting comparison of branch lengths between the RAxML tree and the morphological tree that was constrained to the RAxML topology (Fig. 6B).

## DISCUSSION

### Has RAD-seq progressed our knowledge of the broad-brush phylogenetics of Ophrys?

RAD-seq is a comparatively recent DNA nucleic acid sequencing approach (e.g. Davey *et al.*, 2013; MacCormack *et al.*, 2013; Wagner *et al.*, 2013; Massatti *et al.*, 2016; Olson *et al.*, 2016; Paun *et al.*, 2016). It requires more complex, resource-intensive procedures for data generation and especially for data refining than do traditional candidate gene methods. The technique generates matrices that are rich in phylogenetically informative characters but are also rich in gaps, and incurs a significant proportion of false-positive homology assertions. It is therefore desirable that, in order to justify the considerable analytical effort, RAD-seq should represent a significant improvement on candidate gene matrices built from phylogenetically popular, rapidly mutating genic regions such as ITS and low-copy protein-coding genes in the nucleus, and various intergenic spacers in the plastids.

Certainly, our matrix proved fairly robust to contrasting tree-building algorithms. Our SplitsTree (Fig. 2) and RAxML (Fig. 3) trees show no clear examples of topological incongruence, though in SplitsTree the relative positions of the *speculum* and *bombyliflora* groups appear more ambiguous, and the pairing of *O. scolopax* and *O. picta* may diverge slightly earlier within group H’. The two trees do differ in relative branch lengths in cases where multiple samples of the same macrospecies (and, in two cases, of the same microspecies) have been analysed; such branches are reliably shorter under SplitsTree and thus yield a result closer to that obtained via cloned ITS sequencing by Devey *et al.* (2008). Bootstrap support is high between macrospecies in the RAxML tree, but this outcome should be expected in a large matrix composed of >4000 phylogenetically informative SNPs; it is arguably the comparatively low bootstrap values evident within groups G–I that are more noteworthy than high values elsewhere in the trees.

When previous molecular phylogenies are introduced into the comparison (Fig. 4), it becomes clear that our RAxML tree deviates topologically from the less well-sampled combined ITS plus *trnL–F* tree of Soliva *et al.* (2001) only in its consistently far higher bootstrap values and its ability confidently to place the *umbilicata* group (J) below groups G–I (compare Fig. 4C and E). Our tree also resembles the basal portion of the six-gene low-copy nuclear gene sequence tree of Breitkopf *et al.* (2015), though RAD-seq offers far more credible relationships in the portion of the trees that is distal to the *apifera* group (F) (compare Fig. 4D with E ).

In the ITS tree of Bateman *et al.* (2003) the branch separating the genus *Ophry*s from its closest relatives was an order of magnitude longer than any internal branch within the genus. The present morphological trees also show a proportionately longer branch separating the genus from any credible outgroups (Fig. 5) – a contrast that is enhanced when the morphological characters are constrained to the molecular topology (Fig. 6). The fact that *Ophrys* is a long-branch taxon within subtribe Orchidinae not only renders more difficult the identification of the most appropriate outgroups but also renders less reliable relationships inferred among ingroup members as a result of employing those relatively distant outgroups.

Previous molecular phylogenetic studies have agreed on an early divergence of two major clades within the genus, separating groups B–E from groups F–J (Fig. 4). However, it has proven especially difficult to place with confidence the undoubtedly primitive *insectifera* lineage (A); it has variously been shown to be the earliest diverging lineage within the genus (Devey *et al.*, 2008; Breitkopf *et al.*, 2015), sister to clade B–E (Bateman *et al.*, 2003) or sister to groups F–J (Soliva *et al.*, 2001; Inda *et al.*, 2012). The present study supports the latter placement with 100 % bootstrap support. However, when viewed collectively, various statistical treatments experimentally applied to our RAD-seq matrix together yielded two of the three placements of group A, depending upon precisely how the initial data were filtered. As well as the placement shown in Fig. 3, we also obtained trees where the *insectifera* group was placed as sister to clade B–E, albeit with lower bootstrap support values.

If relative branch length is also taken into account as a classificatory criterion, following the recommendations of Bateman (2009), the present data support subdivision of *Ophrys* into three subgenera, as has long been argued by one of us (R.B.); other observers have recently reached the same conclusion (e.g. Monferrand and Laporte-Cru, 2013; Hennecke, 2016). Comparatively long branches separate macrospecies A (*insectifera*), B–E (*speculum*, *bombyliflora*, *tenthredinifera* and *fusca*) and F–J (*apifera*, *umbilicata*, *sphegodes*, *fuciflora* plus *scolopax*). Groups A–H’ plus J can then be recognized as nine formal sections by taxonomic ‘splitters’ willing to abandon both monophyly and long-term reproductive isolation as criteria for species delimitation, or alternatively they can be viewed by taxonomic ‘lumpers’ as the only nine circumscribable entities sufficiently genetically cohesive to merit recognition as *bona fide* species.

### Is there additional support for the RAD-seq topology from previously published, non-genetic data?

#### Cytogenetics.

Considerable support for our RAD-seq topology can be extracted from the cytogenetics literature, especially the detailed karyotypic observations of D’Emerico *et al.* (2005; see also Cozzolino *et al.*, 2004). Although their study lacked representatives of the *speculum* and *umbilicata* groups, it is nonetheless clear that intrachromosomal asymmetry values (termed A1) fall into three categories that correspond with our three subgenera. The two more extreme values were reported in group A (*insectifera*: A1 >0.36, possessing an especially high ratio of submetacentric to metacentric chromosomes) and groups B–E (A1 <0.29); sandwiched between them were groups F–J (A1 0.29–0.36). In contrast, interchromosomal asymmetry values (termed A2) correlate less well with phylogenetic placement, the *apifera* group having the largest values and the *tenthredinifera* group the smallest. When seeking more precise diagnostic characters, D’Emerico *et al.* (2005) reported a markedly constricted chromosome shared by the *tenthredinifera* and *bombyliflora* groups, and noted especially large satellites on the short arms of the largest chromosome pair in groups F–H’. We perceive this variation in karyotypes as being phylogenetically constrained, rather than reflecting pollinator adaptation as was suggested by Cozzolino *et al.* (2004).

Although D’Emerico *et al.* (2005) and others (e.g. Xu *et al.*, 2011; Sedeek *et al.*, 2014) observed only diploids (2*n* = 36), there have been occasional reports of triploids, tetraploids and pentaploids within the *fusca* group (Greilhuber and Ehrendorfer, 1975; Bernardos *et al.*, 2003) and rare reports of tetraploids within the *umbilicata* and *fuciflora* groups. Surprisingly, the Plant DNA C-values database (Bennett and Leitch, 2012) presently reports no C-values for *Ophrys*, but recent flow-cytometric studies indicate that polyploidy is more frequent within the genus than was previously believed – certainly, triploids (Bianco *et al.*, 1991) and tetraploids (Bateman *et al.*, 2018) occur within the *tenthredinifera* group. Arguably more impressive is the recent discovery that *Ophrys* labella undergo extensive endoreplication, including the poorly understood phenomenon of progressively partial endoreplication recently reported in other genera of subtribe Orchidinae (Travnicek *et al.*, 2015; Hribova *et al.*, 2016); the *Ophrys* labellum is in effect a colony of co-operating cells that differ radically in both ploidy level and primary function (Bateman *et al.*, 2018).

#### Biogeography.

Of the orchid genera native to Europe, only *Ophrys* and *Serapias* are restricted to Europe, Asia Minor and North Africa. Briefly considering the geographic distributions of the nine molecularly circumscribed groups (cf. Delforge, 2006; Pedersen and Faurholdt, 2007), three of the four members of the B–F clade – the *speculum*, *bombyliflora* and *tenthredinifera* groups – are today largely confined to the Mediterranean Basin, suggesting that there exists a phylogenetic constraint on northward migration. The *fusca* (E) and *apifera* (F) groups are similarly widespread west–east but penetrate somewhat further north. The only distributions that lack extensive overlap (i.e. approach allopatry) are those of the *umbilicata* group (J) in the east and the *insectifera* group (A) in the west and north-west, the mountains of Greece and Macedonia apparently defining the border between the two groups. The *fuciflora* group (H) is also traditionally viewed as dominantly western, but its necessary fusion with the former *scolopax* group (I) confers on the aggregate *fuciflora sensu latissimo* group (H’) a more widespread distribution comparable with those of *fusca* and *apifera*. On the basis of these data, there is little chance of confidently identifying the geographic region of origin of the genus, though Breitkopf *et al.* (2015) argued that the most recent common ancestor of the extant lineages did not originate in the east. Considering the other end of the phylogenetic scale, the arrangement of individual accessions within the *fuciflora* (H’) and *sphegodes* (G) groups suggests east to west trends of lineage origination within both groups, which will be explored in greater detail in a future paper (G. Sramkó *et al*., unpubl. res.).

#### Mycorrhizal associates.

Mycorrhizal studies of *Ophrys* have also been surprisingly limited thus far. Current evidence suggests at best only weak host specificity, as was reported by Liebel *et al.* (2010) in representatives of three phylogenetically disparate groups (E, F and H). This situation contrasts with greater mycorrhizal specificity reported from some other genera within subtribe Orchidinae (Jacquemyn *et al.*, 2012; Tesitelova *et al.*, 2013; Bateman *et al.*, 2014). The considerable invasive power shown by some *Ophrys* lineages when rapidly occupying anthropogenically disturbed sites does suggest either a lack of specificity or a lower dependency on mycorrhizae than that observed in many other European orchid genera.

#### Pollinators.

So much has been written during the last century about the charismatic mode of pollination in *Ophrys*, and the pollinating insects that enact pseudo-copulation, that we feel under no obligation to repeat those observations here. It is sufficient to note that the specificity of the relationship between *Ophrys* microspecies and their pollinators is increasingly debated (cf. Bateman *et al.*, 2011; Schlüter *et al.*, 2011; Vereecken *et al.*, 2011; Bateman, 2012; Paulus, 2015). Breitkopf *et al.* (2015) mapped across their low-copy nuclear gene tree the distribution of the three main guilds of pollinators – *Andrena* bees, *Eucera* bees and wasps – and concluded that each of the nine macrospecies has a dominant pollinator guild, wasps being confined to the early-divergent *insectifera* and *speculum* lineages and relationships probably forming between *Ophrys* and *Eucera* bees before those involving *Andrena* bees. However, the summary of pollinator observations presented as their appendix 1 by Claessens and Kleynen (2011; see also their online updates) demonstrates wider spectra of pollinators for most macrospecies – a conclusion more in accord with the now irrefutable body of genetic evidence demonstrating extensive gene flow among two or more microspecies within the nine macrospecies (e.g. Devey *et al.*, 2008; Breitkopf *et al.*, 2013; Sedeek *et al.*, 2014; Cotrim *et al.*, 2016). Admittedly, field observations offer a more diverse spectrum of results, some (e.g. Breitkopf *et al.*, 2013) suggesting more gene flow than others (e.g. Xu *et al.*, 2011). Such contrasts should be expected, given the extremely short-term and geographically localized nature of such studies to date.

### ‘Subgenus *Pseudophrys’* is evolutionarily derived but paedomorphic

Our RAD-seq results make even clearer the already well-known fact that Godfery’s (1928) ‘Subgenus *Pseudophrys*’ (our *fusca* group, E) – a taxon still frequently employed in specialist orchid literature – cannot be sustained at that rank. Given its derived position in the RAD-seq trees, continued recognition as a subgenus would render paraphyletic the remainder of the genus *Ophrys* (i.e. the so-called ‘subgenus *Euophrys*’).

The first three lineages to diverge in our morphological cladistic analyses are, successively, *fusca*, *insectifera* and *speculum* (Fig. 5). Certainly, the *fusca* and *insectifera* groups share considerably more character states with the three outgroup taxa than do any of the other seven *Ophrys* lineages (Table 3). Although the shared plesiomorphic states include having a labellum that possesses well-developed lateral lobes plus a bilobed central lobe, the majority of the shared states reflect comparative simplicity. The *fusca*, *insectifera* and *speculum* groups have less three-dimensional labella that lack a discrete appendix, develop comparatively simple markings and, when viewed under the microscope, exhibit comparatively few epidermal cell types. Comparison with the rooted RAxML tree (Fig. 3) immediately demonstrates that the morphological simplicity of these three lineages is of two very different kinds. The *insectifera* and *speculum* groups are indeed comparatively primitive, representing relatively early evolutionary steps on the road to the more complex floral morphologies that are routinely shown by the more evolutionarily derived groups. In contrast, the logic of parsimony dictates that the simplicity of the *fusca* group flowers is secondarily derived from a more complex common ancestor.

Both the present RAD-seq study and the low-copy nuclear sequence trees of Breitkopf *et al.* (2015) actually place the *fusca* group as sister to *tenthredinifera*, the pair together constituting the most derived portion of the B–E clade (Figs 3 and 4). This is the most morphologically incongruous relationship depicted in the RAD-seq trees; the morphological characters linking these two groups are both few in number and homoplastic in nature, representing the loss of features found in the earlier-diverging *speculum* and *bombyliflora* lineages (Fig. 6). More encouragingly, the characterization of osmophores in clade B–E by Francisco *et al.* (2015) revealed apparent synapomorphies of *fusca* and *tenthredinifera* in the form of an osmophore that occupied the surface of the apical region of the labellum but was of restricted extent on the adaxial surface (Francisco *et al.* also identified in the osmophores of *fusca* potential autapomorphic states). Unsurprisingly, shifting the *fusca* group from its morphologically determined primitive position to its molecularly determined derived position greatly increases perceived homoplasy among the morphological characters across the tree.

Such a radical incongruence between phenotypic and genotypic data sets demands more detailed exploration. Comparison of relative lengths of branches internal to the genus *Ophrys* between the RAxML tree (Fig. 3) and constrained morphology tree (Fig. 6A) failed to reveal a statistically significant positive correlation (Fig. 6B). However, we note that the *fusca* group (E) is subtended by a relatively long branch in both the molecular and constrained morphology trees. This positive correlation in the case of this particular branch indicates an accelerated rate of evolution within the *fusca* lineage. It is tempting to attribute this large number of character-state changes to that most nebulous of aggregate processes, ‘selection pressures’ – for example, one possible driver of these numerous character-state changes would be a switch from a dominantly cephalic to a dominantly abdominal mode of pollination. However, the loss of several features and resultant morphological simplification of the *fusca* flower suggests a possible evolutionary–developmental shift that has resulted in the category of heterochronic change that is termed paedomorphosis – retention of ancestrally juvenile phenotypic features in the mature descendant. Paedomorphosis could have arisen through decreased rate of growth (i.e. neoteny), but our limited observations suggest little difference in speed of floral development between the *fusca* group and the remainder of the genus. This conclusion places the emphasis firmly on precocious offset of growth (i.e. progenesis: Alberch *et al.*, 1979; Bateman, 1994) – the development of several elements of the *fusca* flower is hypothesized to terminate earlier than in other groups of *Ophrys*. Unfortunately, thus far, observations of the floral ontogeny of *Ophrys* have been few and largely confined to micromorphological features (e.g. Bradshaw *et al.*, 2010), precluding adequate testing of this hypothesis.

### Phenotypic convergence operates at every phylogenetic scale within *Ophrys*

Homoplasy indices for the morphological cladistic matrix are typical for a matrix of 16 scored taxa and 43 characters, but imposing the RAD-seq topology on those characters increased perceived homoplasy by approximately 20 %. Unsurprisingly, dominant lateral sepal colour is the most homoplastic character, changing six times in a tree of only 14 scored taxa – an intriguing outcome in the light of a recent study that suggested *Ophrys* sepal colour is a much stronger influence on pollinator preference than is speculum size and shape (Streinzer *et al.*, 2010). Moreover, although the gynostemial ‘beak’ does indeed, as has often been argued, provide a reliable synapomorphy of the F–J clade, there is not a single non-homoplastic synapomorphy present within that clade (Fig. 6). Rather, phenotypic anarchy dominates beyond that point, indicating extensive reticulation. Character states – most of them evolutionarily and possibly epigenetically malleable – switch backwards and forwards within these molecularly delimited groups as labella become more or less three-dimensional, lateral labellar lobes expand or contract, appendixes become more or less prominent, papillae/trichomes lengthen or shorten in contrasting regions of the adaxial epidermis, specula become more or less complex or shift their position relative to the horizontal plane, and the pink-purple anthocyanin wash within the epidermis of the sepals waxes and wanes.

Overall, morphological divergence is as great among *Ophrys* subgroups (notably the *bertolonii* subgroup within the *sphegodes* group) as between the molecularly circumscribed groups. As is evident in sets of most-parsimonious trees resulting from the unconstrained morphological analyses (Fig. 5), morphological characters alone fail reliably to reconstruct the molecularly determined monophyly of groups H’ and J. Specifically, *scolopax* (group H’) often replaces *bornmuelleri* (group J) as sister to *umbilicata* (group J), whereas *bornmuelleri* associates more frequently with *fuciflora* (group H’). Both of these implied pairwise relationships would resurrect groups that have long been recognized by traditional taxonomy (cf. Delforge, 2006, 2016; Pedersen and Faurholdt, 2007), but are clearly refuted by the RAD-seq data.

The most fundamental difference between the unconstrained and constrained morphological trees is the position of the *tenthredinifera* group as a derived member of the B–F clade in the RAD-seq tree (Fig. 3) but as a derived member of the G–J clade in the unconstrained morphological trees, associating with the *fuciflora* group and the *bornmuelleri* subgroup of the *umbilicata* group (Fig. 5). Only in the NJ tree that retained polymorphic scoring of morphology (not shown) was *tenthredifera* placed below *apifera*. This radical incongruence reflects a high degree of convergent phenotypic evolution (*sensu*Scotland, 2011) between *tenthredinifera* and members of the G–J clade (Fig. 6); similarities evident in Table 3 include a large, discrete appendix, comparatively homogeneous labellar trichomes, a well-defined pale margin to the speculum, short triangular lateral petals and an erect (often obovate) median sepal. Given such morphological convergence, it is not surprising that *tenthredinifera* behaves phylogenetically as a destabilizing ‘wildcard’ taxon. Nonetheless, sufficient evidence has accumulated to dispel the myth that *tenthredinifera* (group B) and *bombyliflora* (group D) constitute a single cohesive, monophyletic group (*contra*Devillers and Devillers-Terschuren, 1994; Delforge, 2006).

### How did the genus originate? Reconstructing the most recent common ancestor of extant *Ophrys* lineages

Having addressed the issues surrounding convergence and paedomorphic simplification, we now focus on the other major question that we wished to address by contrasting morphological characters to a robust molecular topology – which features are most likely to have characterized the first *Ophrys*? This question is actually deceptively complex. We have clearly established that the genus is subtended by a long branch, irrespective of whether it is viewed phenotypically or genotypically. That branch implies a long period of independent evolution of the lineage; molecular dating by Sramkó *et al.* (2014) suggested that the lineage originated at approximately 13 Ma, whereas the much shorter molecular branches separating the extant lineages indicate that their most recent common ancestor originated at 3–4 Ma. Slightly earlier divergence dates of approximately 4.5 Ma and approximately 5 Ma were estimated by Inda *et al.* (2012) and Breitkopf *et al.* (2015), respectively – dates closer to the very brief period when the former salt basin flooded to form the Mediterranean Sea. Whatever the accuracy of these estimates, the most important constraint is that, by definition, the present data are competent only to reconstruct the phenotype of the most recent common ancestor of these extant lineages.

This strong asymmetry in branch lengths before vs. after the most recent common ancestor is unfortunate, as it is clear from the constrained morphological phylogeny (Fig. 6) that the majority of the character states that separate *Ophrys* from its closest extant relatives were acquired during that approximately 10 myr intervening period. As summarized in Fig. 6, these features include seven non-homoplastic synapomorphies that have been retained by all subsequently evolving members of the genus: loss of labellar spur, dominantly pilose adaxial epidermis of the labellum and lateral petals, twin bursicles, a lax inflorescence of few large flowers wherein the buds are protected by foliose rather than membranous bracts, and ventral positioning of the labellum achieved through bud inversion rather than pedicel torsion. Reduction in size of lateral petals and acquisition of at least a rudimentary speculum are also implied.

Long branches are inimical to reconstructing most recent common ancestors, as they prevent determination of the temporal sequence in which those many character-state changes took place (Bateman *et al.*, 2006). It also remains debatable at what point during the acquisition of those numerous synapomorphies we would have recognized the then phenotype of the lineage as representing the genus that today is named *Ophrys*. Identification of potentially key synapomorphies is consequently left in the realm of speculation. It seems likely that at least one of the several pseudo-pheromones evolved during this approximately 10 myr period, their advent eventually leading to pseudo-copulation as the dominant mode of pollination within the lineage but perhaps initially arising through pre-adaptation (Schiestl and Cozzolino, 2008; Vereecken *et al.*, 2012). Bateman (2009) speculated that the often extreme three-dimensional topography of the labellum of the more derived *Ophrys* taxa, which emphasizes adaxially convex projections, could not have evolved without first losing the ancestral spur – a strongly concave feature of the labellum and one that is present in all of the outgroups.

Assuming a fairly constant rate of mutation, a molecular long branch can reflect one of three scenarios: (1) a long period during which the lineage was consistently subjected to gene flow sufficient to prevent lineage divergence; (2) a long period during which lineage divergence occurred but was subsequently masked by gene flow during secondary contact between the formerly independent daughter lineages (i.e. hybridization); or (3) a long period during which lineage divergence occurred but all but one of the independent daughter lineages suffered extinction. Explanation (3) appears to us unlikely in a genus that is renowned for being prone to extensive incipient speciation. Both explanations (1) and (2) fall within the broad banner of reticulation, and thus challenge the dichotomous representations that are the focus of the present paper (cf. Bateman et al., 2003, 2011; Devey *et al.*, 2008). We hope that ongoing deeper exploration of extended RAD-seq data, focusing on the F–J clade, will help us to distinguish between hypotheses (1) and (2) (G. Sramkó *et al*., unpubl. res.; R. M. Bateman *et al*., unpubl. res.).

In earlier molecular phylogenies that placed *insectifera* as the single earliest divergent extant lineage within *Ophrys* (Devey *et al.*, 2008; Breitkopf *et al.*, 2015), this species strongly influenced our concept of the likely appearance of the most recent common ancestor of the extant lineages. Potentially primitive character states that comfortably fit prior expectations of plesiomorphy include its comparatively elongate, two-dimensional labellum, trilobed and with a notched central lobe lacking an appendix, a simple isolated speculum and a relatively undifferentiated basal region that includes a basal field enclosed by labia. The absence of long trichomes (reflecting an overall comparative poverty of epidermal cell types), the short, blunt gynostemium and forward-directed median sepal also fit well prior expectations of plesiomorphy (though, as already discussed, these expectations have been determinedly refuted in the case of the *fusca* group).

However, in the present phylogenetic reconstruction, the *speculum* group has gained status comparable with that held by *insectifera*, as both occupy unshared branches and are only one node removed from the most recent common ancestor. Thus, each has equal influence when reconstructing the phenotype of the hypothetical plant that occupies the underlying node. The *speculum* group possesses a larger proportion of apomorphic character states, but nonetheless does not radically alter the ancestral phenotype suggested by *insectifera*. Other features of the most recent common ancestor remain more difficult to predict, including the size, location, epidermal and marginal features of the speculum, and the size and shape of the lateral petals.

Francisco *et al.* (2015) similarly attempted to reconstruct a most recent common ancestor – in their case, that of the B–E clade only. However, they relied upon a highly unstable topology based only on morphological characters that consistently placed the *speculum* group in an improbably derived position on their cladograms (in their study, rendering the *speculum* lineage earliest divergent required an additional 2.73 steps). They bravely divided floral characters far more finely than either ourselves or Devillers and Devillers-Terschuren (1994), seeking sufficient characters to resolve relationships fully among groups B–E, but the consequent need to proliferate into numerous smaller-scale characters – especially those summarizing epidermal micromorphology – carries additional risks of both pleiotropy and misidentification of primary homologies.

Nonetheless, such characters have been utilized to varying degrees in all morphological cladistic analyses of the genus thus far attempted. They actually lie less comfortably in rooted phylogenetic trees operating within the realm of classical taxic homology and more in the much greyer area variously termed homiology, latent homology or underlying synapomorphy – in other words, in the expression of developmental genes heavily mediated (and often masked) by a wide range of epigenetic, ontogenetic and ecophenotypic influences. The recent identification of extensive endoreplication in *Ophrys* flowers (Bateman *et al.*, 2018) can only reinforce such concerns. In the absence of detailed ontogenetic studies, the presumed positional non-homology of characters such as inner versus outer labia and temporal versus staminodial calli also remains suspect.

### Will post-RAD genetic techniques provide deeper insights into phylogeny within *Ophrys*?

Sufficient molecular techniques have now been applied to *Ophrys* at a sufficiently wide range of demographic levels (i.e. from genus-wide studies such as this through to detailed examinations of single populations) to warrant a review of not only what has been learned about their genetics but also what has been learned about the relative merits of the contrasting analytical methods.

It is important first to review briefly the context of *Ophrys* genetics. The genus has a moderately large haploid genome size of approximately 10 pg (Leitch *et al.*, 2009; Bateman *et al.*, 2018). Almost all taxa rely for reproduction entirely on bee/wasp-mediated allogamy. The only known exceptions to this generalization are *O. apifera*, whose unusually slender caudicles permit facultative autogamy (and thereby an exceptionally high fruit set averaging 78 ± 18 %: Claessens and Kleynen, 2011) and *O. bombyliflora*, whose subterranean stolons permit rapid clonal expansion of some local populations. Flowers per inflorescence are few throughout the genus; moreover, fruit set typically averages <25 %, and often <10 % (Claessens and Kleynen, 2011). The twin bursicles that enclose the viscid discs terminating the pollinaria – a feature found throughout *Ophrys* – contrast with the single bursicle that characterizes the outgroup genera, and may constitute an adaptation to encourage removal of at least one of the two pollinaria.

Having placed heavy reliance on pre-zygotic isolation through attracting limited spectra of pollinating species (e.g. Cozzolino and Widmer, 2005; Scopece *et al.*, 2007), *Ophrys* has at best only weak post-zygotic isolating mechanisms, as evidenced by frequent natural hybrids (e.g. Danesch and Danesch, 1972; Bateman *et al.*, 2011). Further evidence is provided by semi-artificial (Xu *et al.*, 2011) and artificial crossing experiments that failed to identify substantially reduced fertility (Scopece *et al.*, 2007; Malmgren, 2008). Any failure of pollinator specificity opens the way for extensive gene flow. Moreover, several features of *Ophrys* conspire to increase greatly the probability that the hybrid progeny of very few interspecific pollinations could successfully dominate a local population in a single generation. These include often maintaining populations of few plants, reliably producing few flowers per individual, having low fruit set, bearing comparatively large flowers each generating several thousand ovules (of which at least 1000 are typically fertile: Bateman *et al.*, 2011; Molnár, 2011) and receiving delivery of numerous pollen grains *en masse* as pollinaria. The key question thus becomes at what frequency gene flow gives sufficient cohesion to a lineage to prevent *bona fide* speciation.

It is also important to recall that the amount of molecular divergence within *Ophrys* is less than that found in any other genus of tribe Orchideae other than *Serapias* (Bateman *et al.*, 2003; Tang *et al.*, 2015). The scale of molecular divergence among the majority of the nine *Ophrys* groups (A–J) may appear substantial in relative terms in Figs 2 and 3, but it is low in absolute terms. Thus, the far smaller molecular disparities among microspecies within the nine groups can only be viewed as negligible. Any analytical technique capable of offering discrimination therefore merits serious consideration.

Among individual genic regions commonly used for phylogeny reconstruction, ITS was the first to be applied to *Ophrys* and remains the region of choice, despite the frequent presence of multiple ribotypes within individual plants revealed by the ITS cloning study of Devey *et al.* (2008). ITS successfully discriminated between the nine major groups recognized in the present RAD-seq study. By concatenating sequences for six low-copy nuclear genes, Breitkopf *et al.* (2015) achieved the same topology as our RAD-seq trees for relationships among the more disparate groups, but they were unable to discriminate between the less disparate *umbilicata* (J), *sphegodes* (G) and *fuciflora–scolopax* (H’) groups (Fig. 4). Earlier, a molecular phylogeny based on only a single low-copy gene, *LEAFY*, had failed to discriminate adequately among microspecies within the *fusca* group (Schlüter *et al.*, 2007). Plastid regions, whether single (*trnL–F* in Soliva *et al.*, 2001; *rpl16* in Inda *et al.*, 2012) or concatenated (Devey *et al.*, 2008), have yielded strongly statistically supported – but most probably incorrect – topologies among the more molecularly divergent lineages, and have reliably failed to discriminate among the less divergent groups F–J.

How can we explain the relative levels of phylogenetic accuracy inferred for these contrasting genic regions? It seems likely from first principles that plastid regions will fall victim to the plastid capture that is a probable consequence of the absence of intrinsic sterility barriers in *Ophrys* and the consequent gene flow that occurs among all members of the genus whenever they come into close proximity. Mitochondrial regions have proven utterly inadequate for within-family phylogeny reconstruction, not just among orchids (cf. Inda *et al.*, 2010) but also among flowering plants in general. The better performance at low taxonomic levels of the approximately 670 bp ITS1–5.8S–ITS2 assembly compared with six concatenated low-copy genes totalling approximately 5350 bp seems most readily attributable to more rapid coalescence (*sensu*Donnelly and Tavaré, 1995). Far from being a phylogenetic hindrance, the existence of multiple copies of ITS – essentially competing with each other for dominance within the relevant lineage – appears to allow ITS to reflect a lineage divergence event more rapidly than can be achieved by multiple low-copy regions (i.e. ITS inherently possesses a shorter ‘molecular divergence lag’ *sensu*Bateman, 2011).

However, even the most passionate advocate for the advent of a candidate gene-based field sequencer (Bateman, 2016) would have to admit that its application to *Ophrys* would be unlikely to bear irresistible fruit. The major lineages within *Ophrys* that are differentiable using the most rapidly mutating nuclear and plastid regions are easily distinguished via traditional morphology alone, whereas the multitude of microspecies thought by some to occur within group E (*fusca*) and groups G–J (i.e. most of the vast panoply of Linnean binomials that infest and ultimately trivialize the genus) cannot be distinguished even when using ITS sequences. It remains to be seen whether any of the genome fragmentation techniques that include RAD-seq and are collectively termed next-generation sequencing (Harrison and Kidner, 2011; Lemmon and Lemmon, 2013; Olson *et al.*, 2016) can be fine-tooled for the identification of at least a minority of the myriad *Ophrys* microspecies. The fact that individuals of the same microspecies show levels of divergence resembling those of different microspecies occurring within the same macrospecies suggests that the resolving power of genetic divergence may, in practice, have reached its natural conclusion with RAD-seq and related techniques. This important issue will be addressed in the next paper in this series, which will apply RAD-seq to a wide selection of microspecies more densely sampled from among the F–J macrospecies (G. Sramkó *et al*., unpubl. res.).

### Conclusions

(1) To the best of our knowledge, this study of the genus *Ophrys* is the first publication to use next-generation RAD-seq to explore phylogenetic relationships among a group of orchids. Topology and branch lengths together reveal three major clades, two of which dichotomize into four minor clades each. Overall, our RAD-seq topology of nine groups (A–H plus J) accords well with past molecular work, and differences among studies can be explained in terms of the taxon sampling and analytical techniques employed. Past karyotypic data also fit the present topology well.
(2) Several areas of incongruence or poor resolution are evident between the RAD-seq trees and topologies generated from our morphological cladistic matrix, which admixes lineages of the three major RAD-seq clades into a paraphyletic series below a poorly resolved ‘crown’. Constraining the morphological characters to the RAD-seq topology adds considerably to perceived levels of homoplasy, suggesting that morphological evolution has been especially non-parsimonious within *Ophrys.*
(3) The inferred homoplasy reflects extensive phenotypic convergence in many of the floral characters plus some losses of character states. Losses are most frequent in the *fusca* lineage, which is inferred to have originated through radical paedomorphic simplification. The molecularly determined derived nature of the *fusca* group provides a superb illustration of (*a*) why simplicity cannot *a priori* be equated with plesiomorphy, and (*b*) why morphological similarity alone is an inadequate basis for evolutionary classification (e.g. Bateman, 2009, 2012). Godfery’s (1928) ‘Subgenus *Pseudophrys*’ can safely be consigned to the dustbin of taxonomic history.
(4) Convergence in floral morphological characters occurs at every phylogenetic level. Unsurprisingly, it is most frequent within the nine macrospecies and among the least disparate clades (i.e. *umbilicata* versus *fuciflora s.l.* versus *sphegodes*), but is also evident between the strongly disparate *tenthredinifera* and *fuciflora* groups. Current evidence is insufficient to determine whether the convergent character states originated within contrasting lineages, most probably through adaptation, or were transferred from one lineage to another through lateral gene transfer.
(5) Using our molecularly constrained morphological matrix to reconstruct the most recent common ancestor of the extant lineages of *Ophrys* has narrowed the range of possible phenotypes likely to have been exhibited by that pivotal plant or population. However, most of the morphological (and molecular) character-state changes that circumscribe the genus occurred before the most recent common ancestor had evolved. Breaking up that all-important sequence of character-state acquisitions will continue to prove extremely challenging in the absence of relevant fossils, though it is possible that ontogenetic and evolutionary-developmental genetic studies could eventually yield indirect evidence.
(6) We can envisage only limited opportunities for future studies to improve the genus-wide characterization of phenotypes within *Ophrys* beyond the data summarized in our Table 3. The vegetative conservatism evident in the genus effectively confines useful variation to floral characters. A very few additional characters could potentially emerge from examining pollinaria in greater detail across the genus, and from expanding the osmophore observations of Francisco *et al.* (2015) from groups B–F to encompass the remaining groups of *Ophrys*. A more dynamic study of floral morphology through ontogeny might also prove informative, as might genus-wide comparisons of biochemistry and chromosome structure.
(7) Similarly, it is possible that RAD-seq may approximate the limit of the resolution that can ever be provided by DNA-based approaches to either phylogeny reconstruction or species circumscription. The ongoing replacement of candidate-gene sequencing by next-generation approaches based on genome fragmentation followed by SNP detection will no doubt continue, though it is unclear whether the technically challenging RAD-seq will remain the technique of choice. In the meantime, we hope that forthcoming deeper explorations of our RAD-seq data that focus on the F–J clade (G. Sramkó *et al*., unpubl. res.), and of genome-skimming data for the G clade alone (R. M. Bateman *et al*., unpubl. res.), will help us not only to better resolve relationships in those groups but also to determine whether divergence beyond nine major lineages (*a*) never occurred or (*b*) occurred but has subsequently been obscured by extensive lateral gene flow.
(8) We recognize that the ground on which long-standing debates regarding species circumscription and speciation processes within *Ophrys* are conducted has recently shifted away from both quantification of genetic divergence and overly simplistic assumptions regarding pollinator specificity toward more sophisticated models and better integrated experimental systems (e.g. Breitkopf *et al.*, 2013; Sedeek *et al.*, 2014). *Bona fide* speciation events within *Ophrys* are likely to reflect small genetic or epigenetic, individual or population-level changes that impact meaningfully on the way that the population interacts with its immediate environment and the other organisms that occupy that environment. It is inevitable that modifications of pseudo-pheromone cocktails will be foremost in the minds of some experienced observers, but many other credible evolutionary scenarios await detailed exploration.
(9) Lastly, from a taxonomic perspective, we remain highly sceptical that Delforge’s (2016) 29 traditionally delimited groups of *Ophrys* can each legitimately be shoehorned in its entirety into our nine monophyletic groups if the monophyly of those nine groups is to be maintained. The case has not been helped by the proliferation of formal names within this charismatic genus, which continues unabated. For example, 102 formal epithets have been added to his European orchid monograph by Delforge between the third (2006) and fourth (2016) editions, making a grand total of 353 *Ophrys* ‘species’ (approx. 20 species less than the figure predicted through extrapolation in fig. 6 of Bateman, 2012). Rather, each named microspecies will need to be rigorously scientifically tested through population genetic, morphometric and ethological study. Only then can groups established previously, through authoritarian pronouncement, be adequately circumscribed and thereby disassembled for evidence-based reallocation to the nine reliably recognizable, monophyletic macrospecies unequivocally delimited here.

## SUPPLEMENTARY DATA

Supplementary data are available at https://academic.oup.com/aob and consist only of Figure S1: Bayesian majority rule tree generated via MrBayes 3.2.2 from the same RAD-seq matrix that was used to generate the RAxML tree shown as Fig. 3.

Supplementary Data

## FUNDING

This work was supported by the Aktion Austria–Hungary (AÖU project 86öu4), the Hungarian Orchid Society, the Hungarian National Research Fund (OTKA PD109686, awarded to G.S.) and the Austrian Science Fund (FWF Y661-B16, awarded to O.P.).
